# Greater interpersonal distance in adults with autism

**DOI:** 10.1002/aur.3013

**Published:** 2023-09-01

**Authors:** Martina Fusaro, Valentina Fanti, Bhismadev Chakrabarti

**Affiliations:** ^1^ Social Neuroscience Laboratory Fondazione Santa Lucia Rome Italy; ^2^ Department of Psychology Sapienza University of Rome Rome Italy; ^3^ Centre for Autism, School of Psychology and Clinical Language Sciences University of Reading Reading UK; ^4^ India Autism Center Kolkata West Bengal India; ^5^ Department of Psychology Ashoka University Sonepat Haryana India

**Keywords:** adults, immersive virtual reality, interpersonal distance, autism

## Abstract

Social interactions are often shaped by the space we prefer to maintain between us and others, that is, interpersonal distance. Being too distant or too close to a stranger can often be perceived as odd, and lead to atypical social interactions. This calibration of appropriate interpersonal distance thus constitutes an important social skill. Individuals with autism spectrum disorder (ASD, hereafter autism) often experience difficulties with this skill, and anecdotal accounts suggest atypical interpersonal distances in their social interactions. In the current study, we systematically measured interpersonal distance in individuals with autism using immersive virtual reality (IVR) to recreate a naturalistic interaction with a full body avatar of a similar age. Participants observed their own virtual body in first‐person perspective, and the other avatar in two tasks: in the first task, they approached the other avatar (active), in the second one they were approached by the other avatar (passive). Two groups of neurotypical and autistic adults, performed both tasks. Autistic adults showed greater interpersonal distance when compared to non‐autistic adults. Additionally, the difference between the passive and active conditions was smaller for non‐autistic compared to autistic adults. Across the full sample, greater interpersonal distance was associated with higher autism‐related traits. This study provides systematic evidence for greater interpersonal distance in autistic adults using a paradigm with high ecological validity and can be useful in informing the design of appropriate environmental adjustments for shared spaces.

## Lay Summary

Social interactions are often shaped by the space we prefer to maintain between us and others, that is, interpersonal distance. We used immersive virtual reality to measure interpersonal distance, where participants either approached or were approached by a virtual character. All participants preferred larger interpersonal distances when they were approached versus when they approached themselves. Autistic adults preferred a larger interpersonal distance compared to non‐autistic adults. These results can inform the design of appropriate environments (e.g., public spaces) for autistic individuals.

## INTRODUCTION

The need to maintain bonds between individuals is intrinsically coupled with the need to regulate the interpersonal distance (IPD) between them, this is particularly evident in the recent context of the global pandemic (Fusaro et al., 2021; Lisi, Scattolin, et al., 2021). The space that surrounds us is key for shaping social interactions (Graziano & Cooke, 2006) and the intrusion of our personal space by another individual may cause feeling of discomfort and anxiety (Hayduk, 1978), resulting in a perceived threat for our physical and mental self. Autism is often characterized by difficulties in social interactions (American Psychiatric Association, 2013). Anecdotal accounts report issues with regulating personal space in autistic individuals. Few studies have followed up these anecdotal accounts, with mixed findings, as pointed out in a recent review conducted by Candini et al. (2020). In a study conducted by Kennedy and Adolphs (2014), the authors reported that, both in children and adolescents through parent reports and in a group of high‐functioning autistic adults through an experimental task, that social distance norms were often violated or altered and persist over a wide age range. In another study by Asada et al. (2016), a group of adolescents had to walk toward the experimenter (i.e., active condition) or look at the experimenter walking toward them (i.e., passive condition). Reduced interpersonal distance was noted in autism compared to neurotypical controls, when there was no eye‐contract between the experimenter and the participant. An opposite pattern of results was noted by Gessaroli et al. (2013), using a similar active‐passive paradigm, where autistic children were more comfortable maintaining a greater distance compared to the neurotypical children. A recent study by Massaccesi et al. (2021) investigated the neurophysiological alterations associated with interpersonal distance processing in autistic individuals. Similar to Gessaroli and colleagues, their study showed that autistic individuals preferred larger interpersonal distance, and that this preference was accompanied by a reduced activity in brain regions found to be involved in the regulation of interpersonal space (bilateral dorsal intraparietal sulcus and left fusiform face area) as well as reduced functional connectivity between these areas and the amygdala. In another study, Perry et al. (2015) investigated interpersonal distance preferences in a group of individuals with autism, using both behavioral measures and electroencephalography. Results showed greater variance in preferred interpersonal distance in the autism group than in the control group, depending on the social anxiety of participants.

Overall, these experiments used an experimenter/confederate in real life as the stimuli. While such an implementation has high ecological validity, it also has potential for greater variability due to greater degrees of freedom (e.g., facial expression, eye gaze, speed of the confederate which cannot be fully controlled, as well as factors such as gender and age of the confederate vis‐à‐vis the participant). To minimize such variability, immersive virtual reality (IVR) offers a powerful tool that can recreate a virtual scenario closely mimicking the real world. Iachini et al. (2014) investigated comfortable distance (active and passive) in a non‐autistic sample using IVR. This study revealed that the distance was modulated by the gender and the age of participants and concluded that IVR studies confirmed findings from real‐world behaviors. Most research on IPD in autism has been conducted on children, and very little is known about IPD in adults. To fill this gap, the current study aimed at comparing IPD and its regulation in autistic and non‐autistic adults. Moreover, differently from Iachini et al. (2016) study, we added the possibility to observe their own, gender‐matched, virtual body in first‐person perspective (Fusaro et al., 2021) in order to enable more realistic interactions (Gorisse et al., 2017). Individuals typically tend to prefer a larger distance when approached by another person/avatar. Hence, we expect to find overall greater distance in the passive compared to the active condition. Considering previous findings, we expected that autistic adults compared to their non‐autistic counterparts would prefer overall greater interpersonal distance with the avatar.

## METHODS

### Participants

Thirty neurotypical subjects (19 females, mean age 26 years old, SD 11; range 18–53 years old) and 23 autistic individuals (13 females, mean age 38 years old, SD 13; range 20–59 years old) were recruited. NT subjects were recruited from within the campus of University of Reading. The individuals with autism were recruited through the Centre for Autism Research Volunteer Panel. One neurotypical adult and two autistic adults were excluded from the analysis due to technical issue with the IVR equipment.

Ethical approval for the study was obtained from the Research Ethics Committee of the University of Reading and all the participants provided informed consent. Along with the main task, participants completed the Autism Spectrum Quotient (AQ; Baron‐Cohen et al., 2001), and were assessed for IQ (see Table 1) using the Wechsler abbreviated scale of intelligence (WASI; Wechsler, 1999).

**TABLE 1:** Demographics, Wechsler abbreviated scale of intelligence (WASI) and autism spectrum quotient (AQ) of the study sample.

Participants	Group	Sex	Mean age (years) (SD)	Range of age	Mean WASI (SD)	Mean AQ (SD)
30	NT	19 F	26, SD 11	18–53	109, SD 13	19.51, SD 5.64
23	Autism	13 F	38, SD 13	20–59	115, SD 11	33.90, SD 6.01

### Experimental stimuli

**FIGURE 1:**
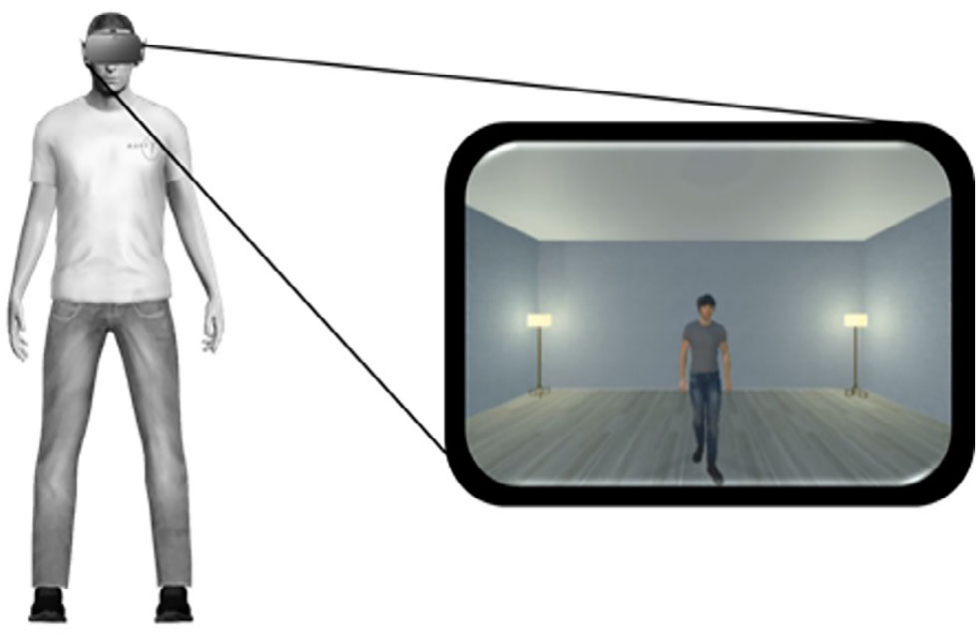
The participant, on the left side of the figure, could observe in the head mounted display a room and an avatar placed in front of him/herself.

The virtual scenario was designed using 3DS Max 2017 (Autodesk, Inc.) and implemented in Unityv5.3.The virtual avatars were created using Iclone 7 (https://www.reallusion.com/iclone/) and implemented in Unity. The scenario was presented by means of a head mounted display (Oculus Rift). The virtual scene consisted of a real‐size room with two avatars standing facing each other. Both the first‐person (1PP) and third‐person (3PP) avatars were the same gender (Figure 1). Participants could approximately adjust the physical size of the other avatar, to match their own. This manipulation was implemented to minimize potential confounds due to dominance (if the other avatar was considerably larger, that can in principle affect interpersonal distance).

### Experimental procedure

The experiment was composed of two blocks, each with three trials. The first block administered to the participants was the “active” condition. After wearing the head mounted display, each participant was asked to have a look at the virtual scene and to two avatars in the scene to familiarize with them. Then the participants were asked to walk toward the 3PP avatar to reach the distance that she or he would generally keep with another person. The distance between the two avatars (measured as the distance between participant's nose and 3PP avatar's nose) recorded when the participant decided to stop walking toward the 3PP avatar. In the “passive” condition, participants were asked to stay still while the 3PP would slowly walk toward them. The participants were instructed to say “Stop” when the 3PP avatar reached the distance that they would usually keep with another person. It is worth noting that in the two conditions the “stop” occurred in two different ways. In the active condition, participants stopped walking toward the avatar when it felt too close to be comfortable. In the passive condition, they would have had to verbally indicate “stop” to make the avatar stop (which was implemented by the experimenter sitting with the controls).

## RESULTS

Interpersonal distance was measured in adults with and without autism under both active and passive conditions. The mean of the three trials in each condition was used to run the analysis. A mixed analysis of variance (ANOVA) was conducted on the measure of the distance in meters, with group (Autism and NT) as a between‐subject variable and Condition (active and passive) as a within‐subject variable.

**FIGURE 2:**
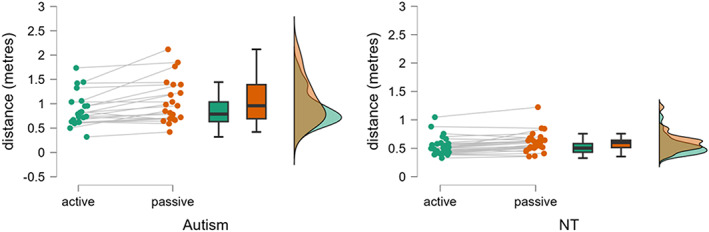
The comparison of interpersonal distance between the passive and active conditions in autistic and non‐autistic participants. The distance is expressed in meters.

A main effect of Group was noted [*F*(1,48) = 24.77; *p* < 0.0001, *ƞ* ^2^ = 0.340], revealing that the interpersonal distance was larger in the Autism group compared to the NT one (NT = mean 0.56 meters (m); standard deviation (sd) ± 0.16, confidence interval (CI) 0.463–0.679; Autism = 0.96 m ± 0.41, CI = 0.846–1.092). A significant main effect of the Condition was also found [*F*(1,48) = 29.091; *p* = 0.001, *ƞ* ^2^ = 0.353], showing larger distance in the passive compared to the active condition (0.79 m ±0.39, CI = 0.702–0.886; 0.67 m ± 0.30, CI = 0.606–0.753, respectively). An interaction Condition × Group was found [*F*(1,48) = 5.443; *p* = 0.024, *ƞ* ^2^ = 0.066]. Post‐hoc analysis showed that IPD in the passive and the active conditions were different in both the NT (active: 0.53 m ± 0.15, CI =0.437–0.629; passive: 0.60 m ± 0.17, CI = 0.482–0.724, *p* = 0.02) and the Autism group (active: 0.88 m ± 0.35, CI = 0.768–0.994; passive: 1.05 m ± 0.45, CI = 0.915–1.199, *p* < 0.001) and that both the active and the passive condition were larger in the Autism than in the NT (*p* < 0.001for both comparisons) (Figure 2). For completeness, an additional ANOVA considering the gender and group as a within‐factor was run but no differences were found between males and females (*F*(1,45) = 0.729, *p* = 0.398) nor the interaction with Group (*F*(1,45) = 1.048, *p* = 0.311) nor with distance (*F*(1,45) = 0.01, *p* = 0.899).

We computed the difference in IPD between active and passive conditions as a delta value (active‐ passive).   A between‐groups *t*‐test on this delta value, between autistic and non‐autistic participants was found to be significant, *t*(48) = −2.333; *p* = 0.024. This comparison is equivalent to our interaction between Condition and Group.

Lastly, we conducted an independent samples *t*‐test on age using Group as grouping variable: *t*(48) = 4.069, *p* < 0.001. We then conducted our ANOVA using “age” as a covariate to see whether this difference could impact our results. Results showed that the effect of condition and group, as well as their interaction remained significant. The main effect of Age was not significant *F*(1,47) = 1.007, *p* = 0.321, nor the interaction between age and condition *F*(1,47) = 2.580, *p* = 0.115.

**FIGURE 3:**
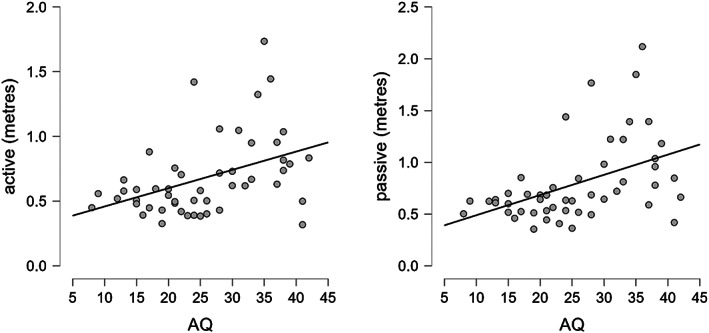
Correlations between AQ and interpersonal distance in active and passive conditions (distance in meters).

Correlation between the two conditions (active and passive) and the AQ was performed across the entire sample. A positive correlation with both active and passive condition was found across the two groups (active: *r* = 0.422, *p* = 0.002; passive: *r* = 0.457, *p* < 0.001) suggesting that higher autism traits were reflected in higher interpersonal distance (Figure 3).

## DISCUSSION

The aim of the present study was to investigate interpersonal distance in autistic and non‐autistic adults. IVR was used as a tool to recreate a naturalistic interaction with an avatar of the same gender and similar age and size, overcoming the limitations that occur while interacting with a real‐life experimenter or one that is significantly different in age from the participant. The main findings of the study indicate that (a) both groups showed larger distance in the passive condition, confirming that people prefer keeping larger interpersonal distances if other people are approaching them (b) autistic adults tended to keep larger distance between themselves and others in both passive and active conditions; and that (c) higher autistic traits correlated with greater distance in both conditions. The difference in IPD between active and passive conditions was larger for autistic adults than for the non‐autistic group.

The observation that both groups preferred to keep a larger distance in the passive condition, is consistent with previous findings (Candini et al., 2017; Iachini et al., 2014). When someone is approaching us, we have less control over the situation, and we cannot predict the intention of the other person. This may cause more insecurity in the person being approached, who prefers greater interpersonal distance in order to feel safe (Graziano & Cooke, 2006). We speculate that in our study, since this result is consistent in both groups, this “protective system” is an ability that is fully preserved also in individuals with autism. It is also worth noting that this need occurs when an object invades our space (Asada et al., 2016) suggesting that the system may not be specific to social stimuli. While both conditions were controlled by the participants' decision, the modalities of control were different—mimicking real life. In the passive condition the reduced sense of agency in the passive condition could therefore have influenced the distance, pushing the participant to stop the avatar in advance. Future studies could investigate whether the speed of approaching or being approached could have an effect on the results (e.g., seeing an avatar that run vs. an avatar slowly walking). Another possible explanation of our results could be found in the motor involvement during the active condition, which may have played a role in the judgment of the interpersonal space (Delevoye‐Turrell et al., 2011). Indeed, when we actively move, we are more aware of the space that surrounds us, compared to the situations in which we are still.

Adults with autism showed greater interpersonal distance than neurotypicals in both conditions. Greater IPD in autistic adults may reflect heightened salience/threat response to dynamic social stimuli. While this result is consistent with some earlier studies with autistic children (Candini et al., 2017, 2019; Gessaroli et al., 2013; Perry et al., 2015), it is inconsistent with others that showed shorter interpersonal distances in autism (Asada et al., 2016; Kennedy & Adolphs, 2014; Lough et al., 2015). Some of this variation could be driven by experimenter effects such as speed of walking or emotion expression. In our study we minimized potential confounds that can arise from an experimenter who is informed about the task and, or/and about the diagnosis status of the participants (Asada et al., 2016). A recent study investigated IPD in autistic children using IVR by Simões et al. (2020). The authors found that there was a significant positive correlation between the IPD in real and virtual scenarios. Moreover, authors showed that, compared to TD, autistic participants appeared to have wider distributions of results. Importantly however, the paradigm involved children interacting with adult experimenters/avatars in this study, which can influence IPD.

It is possible that greater interpersonal distance may be driven by greater social anxiety, as shown in an earlier study (Perry et al., 2015) or be linked to reduced motivation toward social stimuli (Gray et al., 2018; Hedger et al., 2020). Unfortunately, we do not have direct measures of social motivation and social anxiety to measure their relative contributions. Finally, it is possible that the current results are influenced by our sample, which was restricted to adults with low‐moderate support needs, who may have devised compensatory strategies for minimizing any potential discomfort in social interactions through increasing IPD. A comparison between autistic adults with high and low support needs may help in discerning the role of learned compensation. The positive correlation between IPD and autism‐related traits (measured with the AQ), indicates that overall, irrespective to the diagnosis of autism, social difficulties may drive the need for greater IPD.

The study presents some important limitations. There is a substantial methodological difference between the active and the passive condition (i.e., participants' motor stop in the first and the vocal signal to stop in the latter). This difference might have impacted in the reliability of our data, in particular in the passive condition. However, it is worth considering that if there was a systematic delay in the experimenter's pressing the button in the passive condition, it would have resulted in shorter interpersonal distances (compared to that at the exact moment that the vocal signal was produced). Despite this possibility, we observed a substantially larger interpersonal distance in the passive compared to the active condition. This suggests that if there was no delay in recording the vocal stop signal, then the interpersonal distance in the passive condition would have been even higher. Moreover, the number of participants in our study is small, thus limiting the generalizability of our findings. Finally, we note that the autism group is associated with a greater variance in RT compared to the control group. This observation is in line with several previous case–control studies of autism, across multiple metrics – which have reported higher variability of response s in the autism group (Fulceri et al., 2018; Geurts et al., 2008; Lombardo et al., 2019; Magnuson et al., 2020).

IVR is a promising tool that is high in ecological validity and quick to administer while retaining strong experimental control (Lisi et al., 2021). This paradigm is scalable for investigating related questions in social behavior (e.g., changing the characteristic of the appearance of the avatar or recreating specific social scenarios). Personal space differs between autistic and non‐autistic adults, and this evidence may be reflected in daily social interactions. Translating these insights into the real world through creating appropriate public spaces (e.g., restaurants, public transportation, and schools) has the potential to improve the quality of life for many autistic individuals.

## FUNDING INFORMATION

MF is supported by the Italian Ministry of Health (Grant Sponsor: Ricerca Finalizzata, Giovani Ricercatori 2019, Grant number: GR‐2019‐12369761) and by the BE FOR ERC Grant from Sapienza University of Rome. VF is supported by the European Research Council (Grant number: 865568) and the University of Reading. BC is supported by the Medical Research Council UK (Grant reference: MR/S036423/1) and the European Research Council (Grant number: 865568).

## Data Availability

Anonymised data that support the findings of this study will be available from the University of Reading Research Data Archive (https://researchdata.reading.ac.uk).

## References

[aur3013-bib-0001] American Psychiatric Association . (2013). Diagnostic and statistical manual of mental disorders. American Psychiatric Association. 10.1176/appi.books.9780890425596

[aur3013-bib-0002] Asada, K. , Tojo, Y. , Osanai, H. , Saito, A. , Hasegawa, T. , & Kumagaya, S. (2016). Reduced personal space in individuals with autism spectrum disorder. PLoS One, 11(1), e0146306. 10.1371/journal.pone.0146306 26814479 PMC4729526

[aur3013-bib-0003] Baron‐Cohen, S. , Wheelwright, S. , Skinner, R. , Martin, J. , & Clubley, E. (2001). The autism‐Spectrum quotient (AQ): Evidence from Asperger syndrome/high‐functioning autism, males and females, scientists and mathematicians. Journal of Autism and Developmental Disorders, 31(1), 5–17. 10.1023/A:1005653411471 11439754

[aur3013-bib-0004] Candini, M. , di Pellegrino, G. , & Frassinetti, F. (2020). The plasticity of the interpersonal space in autism spectrum disorder. Neuropsychologia, 147, 107589. 32827540 10.1016/j.neuropsychologia.2020.107589

[aur3013-bib-0005] Candini, M. , Giuberti, V. , Santelli, E. , di Pellegrino, G. , & Frassinetti, F. (2019). When social and action spaces diverge: A study in children with typical development and autism. Autism, 23(7), 1687–1698. 10.1177/1362361318822504 30663321

[aur3013-bib-0006] Candini, M. , Giuberti, V. , Manattini, A. , Grittani, S. , di Pellegrino, G. , & Frassinetti, F. (2017). Personal space regulation in childhood autism: Effects of social interaction and person's perspective. Autism Research, 10(1), 144–154. 10.1002/aur.1637 27157094

[aur3013-bib-0007] Delevoye‐Turrell, Y. , Vienne, C. , & Coello, Y. (2011). Space boundaries in schizophrenia voluntary action for improved judgments of social distances. Social Psychology, 42(3), 193–204. 10.1027/1864-9335/a000063

[aur3013-bib-0008] Fulceri, F. , Tonacci, A. , Lucaferro, A. , Apicella, F. , Narzisi, A. , Vincenti, G. , Muratori, F. , & Contaldo, A. (2018). Interpersonal motor coordination during joint actions in children with and without autism spectrum disorder: The role of motor information. Research in Developmental Disabilities, 80, 13–23. 29879613 10.1016/j.ridd.2018.05.018

[aur3013-bib-0009] Fusaro, M. , Lisi, M. P. , Tieri, G. , & Aglioti, S. M. (2021). Heterosexual, gay, and lesbian people's reactivity to virtual caresses on their embodied avatars' taboo zones. Scientific Reports, 11(1), 2221. 10.1038/s41598-021-81168-w 33500486 PMC7838160

[aur3013-bib-0010] Gessaroli, E. , Santelli, E. , di Pellegrino, G. , & Frassinetti, F. (2013). Personal space regulation in childhood autism Spectrum disorders. PLoS One, 8(9), 74959. 10.1371/journal.pone.0074959 PMC3781155 24086410

[aur3013-bib-0011] Geurts, H. M. , Grasman, R. P. , Verté, S. , Oosterlaan, J. , Roeyers, H. , van Kammen, S. M. , & Sergeant, J. A. (2008). Intra‐individual variability in ADHD, autism spectrum disorders and Tourette's syndrome. Neuropsychologia, 46(13), 3030–3041. 18619477 10.1016/j.neuropsychologia.2008.06.013

[aur3013-bib-0012] Gorisse, G. , Christmann, O. , Amato, E. A. , & Richir, S. (2017). First‐ and third‐person perspectives in immersive virtual environments: Presence and performance analysis of embodied users. Frontiers in Robotics and AI, 4, 33. 10.3389/frobt.2017.00033 PMC7805911 33501025

[aur3013-bib-0013] Gray, K. L. H. , Haffey, A. , Mihaylova, H. L. , & Chakrabarti, B. (2018). Lack of privileged access to awareness for rewarding social scenes in autism Spectrum disorder. Journal of Autism and Developmental Disorders, 48(10), 3311–3318. 10.1007/s10803-018-3595-9 29728947 PMC6153919

[aur3013-bib-0014] Graziano, M. S. A. , & Cooke, D. F. (2006). Parieto‐frontal interactions, personal space, and defensive behavior. Neuropsychologia, 44(6), 845–859. 10.1016/j.neuropsychologia.2005.09.009 16277998

[aur3013-bib-0015] Hayduk, L. A. (1978). Personal space: An evaluative and orienting overview. Psychological Bulletin, 85(1), 117–134. 10.1037/0033-2909.85.1.117

[aur3013-bib-0016] Hedger, N. , Dubey, I. , & Chakrabarti, B. (2020). Social orienting and social seeking behaviors in ASD. A meta analytic investigation. Neuroscience and Biobehavioral Reviews, 119, 376–395. 10.1016/j.neubiorev.2020.10.003 33069686

[aur3013-bib-0017] Iachini, T. , Coello, Y. , Frassinetti, F. , & Ruggiero, G. (2014). Body space in social interactions: A comparison of reaching and comfort distance in immersive virtual reality. PLoS One, 9(11), e111511. 10.1371/journal.pone.0111511 25405344 PMC4236010

[aur3013-bib-0018] Iachini, T. , Coello, Y. , Frassinetti, F. , Senese, V. P. , Galante, F. , & Ruggiero, G. (2016). Peripersonal and interpersonal space in virtual and real environments: Effects of gender and age. Journal of Environmental Psychology, 45, 154–164. 10.1016/j.jenvp.2016.01.004

[aur3013-bib-0019] Kennedy, D. P. , & Adolphs, R. (2014). Violations of personal space by individuals with autism spectrum disorder. PLoS One, 9(8), 103369. 10.1371/journal.pone.0103369 PMC4123873 25100326

[aur3013-bib-0021] Lisi, M. P. , Fusaro, M. , Tieri, G. , & Aglioti, S. M. (2021). Humans adjust virtual comfort‐distance towards an artificial agent depending on their sexual orientation and implicit prejudice against gay men. Computers in Human Behavior, 125, 106948.

[aur3013-bib-0022] Lisi, M. P. , Scattolin, M. , Fusaro, M. , & Aglioti, S. M. (2021). A Bayesian approach to reveal the key role of mask wearing in modulating projected interpersonal distance during the first COVID‐19 outbreak. PLoS One, 16(8), e0255598. 34375361 10.1371/journal.pone.0255598 PMC8354471

[aur3013-bib-0023] Lombardo, M. V. , Lai, M. C. , & Baron‐Cohen, S. (2019). Big data approaches to decomposing heterogeneity across the autism spectrum. Molecular Psychiatry, 24(10), 1435–1450. 30617272 10.1038/s41380-018-0321-0 PMC6754748

[aur3013-bib-0024] Lough, E. , Hanley, M. , Rodgers, J. , South, M. , Kirk, H. , Kennedy, D. P. , & Riby, D. M. (2015). Violations of personal space in young people with autism Spectrum disorders and Williams syndrome: Insights from the social responsiveness scale. Journal of Autism and Developmental Disorders, 45(12), 4101–4108. 10.1007/s10803-015-2536-0 26206231

[aur3013-bib-0025] Magnuson, J. R. , Iarocci, G. , Doesburg, S. M. , & Moreno, S. (2020). Increased intra‐subject variability of reaction times and single‐trial event‐related potential components in children with autism spectrum disorder. Autism Research, 13(2), 221–229. 31566907 10.1002/aur.2210

[aur3013-bib-0026] Massaccesi, C. , Groessing, A. , Rosenberger, L. A. , Hartmann, H. , Candini, M. , Di Pellegrino, G. , Frassinetti, F. , & Silani, G. (2021). Neural correlates of interpersonal space permeability and flexibility in autism spectrum disorder. Cerebral Cortex, 31(6), 2968–2979. 33511981 10.1093/cercor/bhaa404

[aur3013-bib-0027] Perry, A. , Levy‐Gigi, E. , Richter‐Levin, G. , & Shamay‐Tsoory, S. G. (2015). Interpersonal distance and social anxiety in autistic spectrum disorders: A behavioral and ERP study. Social Neuroscience, 10(4), 354–365. 10.1080/17470919.2015.1010740 25666260

[aur3013-bib-0028] Simões, M. , Mouga, S. , Pereira, A. C. , de Carvalho, P. , Oliveira, G. , & Castelo‐Branco, M. (2020). Virtual reality immersion rescales regulation of interpersonal distance in controls but not in autism Spectrum disorder. Journal of Autism and Developmental Disorders, 50(12), 4317–4328. 10.1007/s10803-020-04484-6 32266686

[aur3013-bib-0128] Wechsler, D. (1999). Manual for the Wechsler Abbreviated Scale of Intelligence. Psychological Corporation.

